# A metabolomics approach to evaluate the effect of lyophilization versus oven drying on the chemical composition of plant extracts

**DOI:** 10.1038/s41598-021-02158-6

**Published:** 2021-11-22

**Authors:** Nancy A. ElNaker, Mariane Daou, Michael A. Ochsenkühn, Shady A. Amin, Ahmed F. Yousef, Lina F. Yousef

**Affiliations:** 1 Department of Chemistry, Khalifa University, P.O BOX 127788, Abu Dhabi, UAE; 2 Biology Program, New York University Abu Dhabi, Saadiyat Island, P.O BOX 129188, Abu Dhabi, UAE; 3 Center for Membranes and Advanced Water Technology (CMAT), Khalifa University, P.O BOX 127788, Abu Dhabi, UAE

**Keywords:** Green chemistry, Medicinal chemistry, Process chemistry, Metabolomics, Biochemistry, Chemical biology, Plant sciences

## Abstract

Lyophilization is the “gold standard” for drying plant extracts, which is important in preserving their quality and extending their shelf-life. Compared to other methods of drying plant extracts, lyophilization is costlier due to equipment, material and operational expenses. An alternative method is post-extraction oven-drying, but the effects of this process on extract quality are unknown. In this study, crude extracts from *Arthrocnemum macrostachyum* shoots were compared using three post-extraction drying methods (lyophilization and oven drying at 40 and 60 °C) and two extraction solvents (water and aqueous 50% ethanol). Untargeted metabolomics coupled with chemometrics analysis revealed that post extraction oven-drying resulted in the loss of up to 27% of molecular features when compared to lyophilization in water extracts only. In contrast, only 3% of molecular features were lost in aqueous 50% ethanol extracts when subjected to oven drying. That is to say, ethanol used as a solvent has a stabilizing effect on metabolites and enhances their resistance to thermal transformation in the oven. Collectively, oven-drying of extracts was as effective as lyophilization in preserving metabolites in extracts only when 50% ethanol was used as a solvent. The results presented in this paper demonstrate the value of selecting solvent-appropriate post-extraction drying methods.

## Introduction

Plants produce secondary metabolites and compounds that have applications in the nutraceutical, cosmeceutical, and pharmaceutical industries^1–4^. In pharmacy, plant secondary metabolites such as carotenoids, phenolic compounds, and nitrogen compounds gained interest for their antioxidant, anti-inflammatory and anti-cancer properties^5–7^. In addition, the anti-microbial and anti-viral properties of some plant metabolites is recently being demonstrated in tackling multidrug resistant microbes as well as emerging viral infections, such as SARS-CoV-2^8–10^. Phytochemicals are also used in the food industry as natural preservatives^11^.

With a market expected to reach US$ 9 billion by 2029, interest has continued to mount in the identification of new phytochemicals and optimization of their production processes^12^. Metabolomics analysis is useful for the comprehensive profiling and comparison of metabolites in a biological system, and it is used extensively in plant metabolism and food science research^13,14^. Metabolomics involves the use of various analytical instruments including nuclear magnetic resonance (NMR), gas chromatography-mass spectrometry (GC–MS) and liquid chromatography-mass spectrometry (LC–MS). UHPLC-QToF-MS is the method of choice for high–throughput identification of metabolites and generates data that can be used to compare the metabolome from different biological systems^15–17^.

The implementation of optimized high-quality agricultural and biomass processing practices is a critical step towards enhancing the quality of plants and improving the safety, quality and consistency of the final products^18^. Dried plant extracts, rich in bioactive metabolites, are the main active ingredients used in the preparation of natural products, which are typically marketed in the form of powders or tablets in order to ensure good stability and correct dosage^19,20^. Consequently, optimizing the extraction protocol, which includes the choice of solvent, solvent to biomass ratio, extraction time, and choice of extraction method, are key factors in the manufacturing process of these natural products^21–23^. Most literature available focused on evaluating the effect of extraction protocol in plant extracts. However, the method for drying plant extracts post-extraction is also important. Particularly because recent studies reported that the drying method used to process plant biomass pre-extraction affects their bioactivity and chemical composition^24–30^. Like-wise, the chemical and bioactive properties of plant extracts are also affected by the post-extraction drying method used^21^.

Spray-drying, rotary evaporation, and lyophilization, also known as freeze-drying, are the most commonly used post-extraction drying methods. Spray drying involves the rapid conversion of a liquid solution or slurry into a powder using hot gas. However, there is an overall loss in sample nutritional value, biological yield, and protein degeneration due to the direct impact of the operating temperature, which is typically 80°C^31^. Rotary evaporation is the process of removing solvents from the extracts using moderate heating and low pressure introduced through vacuum. This process is efficient in removing organic solvents, but is not as efficient when drying aqueous extracts^32^. Lyophilization, where the solvent is removed at low temperature through sublimation, is the gold standard used in the pharmaceutical, cosmeceutical, nutraceutical, and chemical industries.

Lyophilization limits oxidative changes of metabolites taking place because the oxygen concentration is deficient under vacuum^33^. However, compared to other methods of drying plant extracts, lyophilization is more costly due to equipment, material and operational costs^34^. For example, the lyophilization of raspberries was reported to consume approximately two times more energy compared to convective drying methods, with an approximately three times longer time required to complete the process^35^. A cost-effective alternative to lyophilization is oven drying, and it typically uses conventional ovens due to low cost and availability of the equipment. Oven drying harvested biomass affects the phytochemical profiles of culinary and medicinal herbs in a temperature dependent manner, where drying at 40 °C resulted in higher total polyphenol content and antioxidant activity compared to fresh samples^36^. The quality and bioactivity-enhancing effect of post-harvest oven-drying at 40 °C was similarly observed in the preparation of *Uraria crinite* herbal tea and *Cistus creticus* dried leaves^30,37^.

Extracts prepared from oven-dried *Arthrocnemum macrostachyum* shoots have previously shown substantial antioxidant potential and high total phenolic content^38–40^. *A. macrostachyum* is a plant that is able to physiologically adapt to hypersaline conditions exceeding 1 M NaCl^41^, and to warm climates in which temperatures exceed 60 °C^42,43^. In traditional medicine, *A. macrostachyum* is used as an antibiotic, alexipharmic and hypoglycemic agent^44–46^. Phytochemical screening of this plant showed high phenol, flavonoid, tannin and alkaloid contents^38^. Despite the health benefits of *A. macrostachyum*, mediated by its antioxidant, enzymes inhibitory and cytotoxic activities (reviewed in^47^), only a few studies evaluated the phytochemical profiles of *A. macrostachyum* extracts using UHPLC- ESI–MS/MS^39,48^. However, those studies only focused the analysis on the ten compounds in methanol extracts that were considered to be the most abundant. In addition, very little is known about the effect of different extraction and drying methods on the metabolomic profile of *A. macrostachyum* extracts.

Even though different studies have examined the effect of post-harvest (pre-extraction) oven-drying on plants^49–55^, to the best of our knowledge, the effect of using this technique to dry plant extracts (post-extraction) was never investigated. Given the bioactivity enhancing effect observed with post-harvest oven-drying and the temperature-dependent variations in the released metabolites, it is important to investigate whether the same outcome can be obtained post-extraction. Therefore, the objective of this paper is to evaluate the effect of this technique, applied at two different temperatures (40 °C and 60 °C), on the metabolomic profiles of 50% (v/v) aqueous ethanol (aq. EtOH) and water extracts of *A. macrostachyum*. To achieve this objective, UHPLC-QToF-MS was used to compare the phytochemical profiles of lyophilized and oven-dried *A. macrostachyum* extracts.

## Results and discussion

### Effect of oven-drying on the metabolomic profiles of *A. macrostachyum* extracts

**Figure 1:**
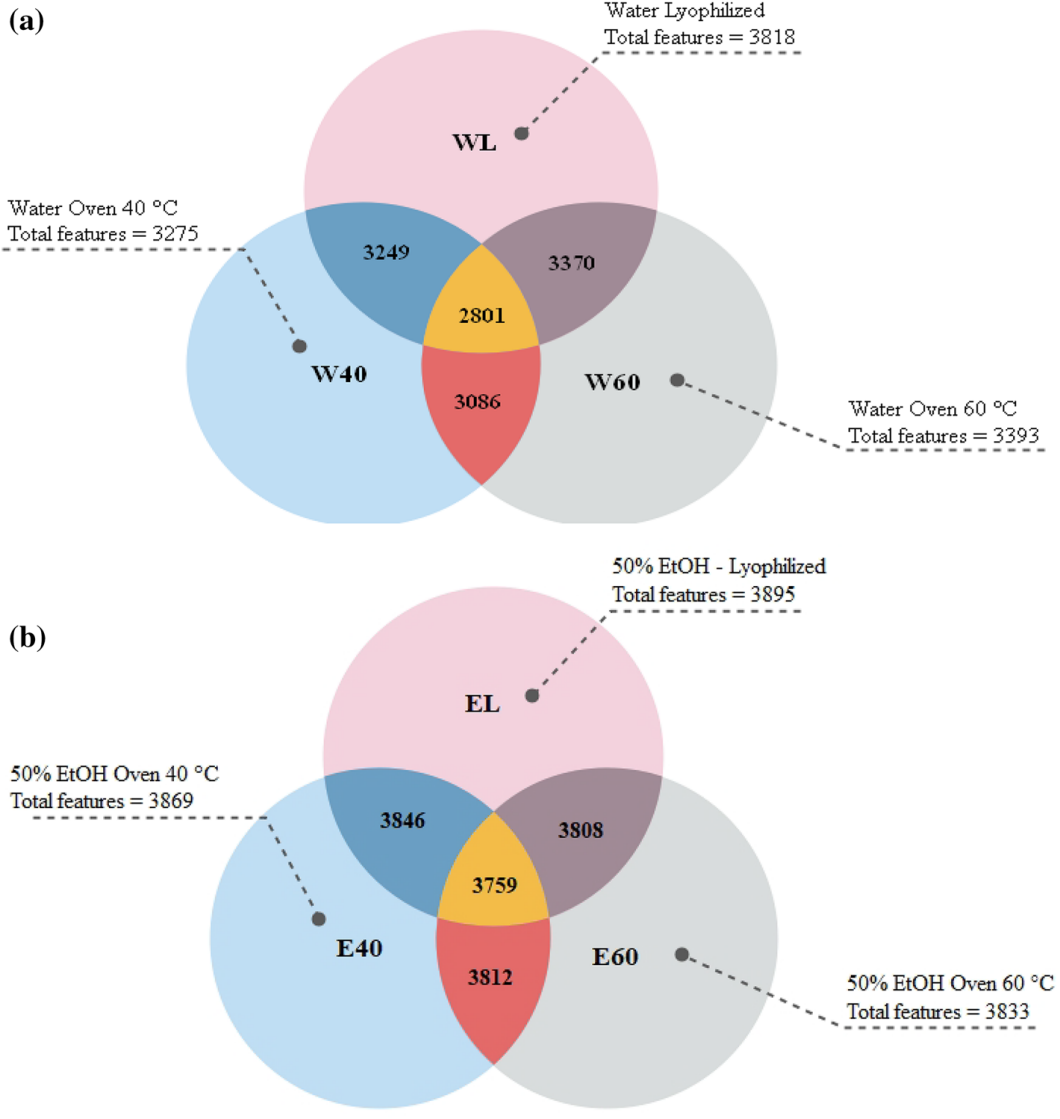
Venn diagram of the detected molecular features using the LC-QToF-MS for *A. macrostachyum* extracts (**a**) water (**b**) 50% EtOH at different drying methods.

The metabolites present in *A. macrostachyum* water and aq. EtOH extracts were investigated after three different post-extraction drying treatments (lyophilization, oven drying at 40 °C or 60 °C). A total of 3984 molecular features were detected in *A. macrostachyum* samples, with more features being identified in the lyophilized samples regardless of the extraction solvent used, indicating a preservation of metabolites using this method (Fig. 1a). Oven drying resulted in the loss of 27% of the detected features in the water extract while only 3% were lost in the aq. EtOH extracts (Fig. 1b). In addition, variations in the molecular features were more pronounced in water extracts compared to aq. EtOH extracts at the two oven drying temperatures used. For example, water extracts that were oven dried at 60 °C had 118 more molecular features compared to extracts dried at 40 °C, while aq. EtOH extracts dried at the different temperatures were more similar, with only 35 more molecular features observed at 40 °C compared to 60 °C. Taken together, this indicates that the drying method and the temperature affects the chemical composition of the extracts, and that ethanol seems to protect the extracted metabolites from thermal degradation when the oven drying method is used.

**Figure 2:**
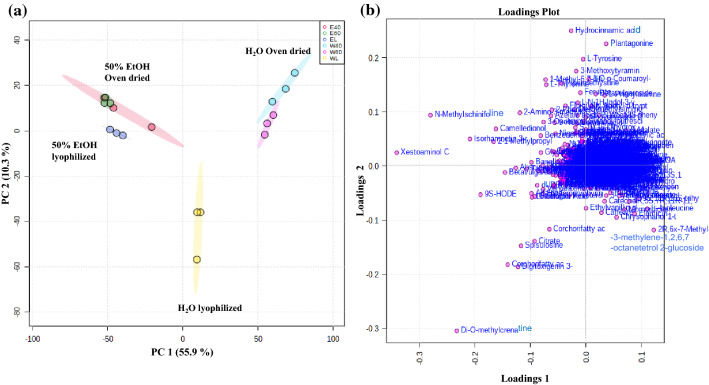
(**a**) PCA score plot derived from positive (ESI+) and negative (ESI−) mode in LC–MS metabolite profiles of *A. macrostachyum* water and 50% EtOH extracts (**b**) PCA loading plots for the selected PCs. Heatmap for the top 50 compounds identified based on PLSDA-VIP scores (distance measure using Euclidean, and clustering algorithm using ward) in water and 50% EtOH *A. macrostachyum* extracts *A. macrostachyum* extracts (**c**) Global features (**d**) Knowns only. WL: lyophilized water extract, W40: oven-dried water extract at 40 °C, W60: oven-dried water extract at 60 °C, EL: lyophilized 50% EtOH extract, E40: oven-dried 50% EtOH extract at 40 °C, E60: oven-dried 50% EtOH extract at 60 °C.

Principal component analysis (PCA) clearly differentiated *A. macrostachyum* extracts based on the extraction solvent used and the post-extraction drying method applied (Fig. 2a). The results showed three distinct clusters: oven-dried water extract, lyophilized water extract and aq. EtOH extracts, with the latter showing lyophilized and oven-dried extracts clustering together. There was a clear discrimination of water *A. macrostachyum* lyophilized extracts from the other processed samples (PC1 = 55.9% versus PC2 = 10.3%). Interestingly, metabolites detected after oven drying or lyophilization of aq. EtOH extracts clustered closer together and the applied oven temperature did not have a pronounced effect in differentiating the extracts as was observed with the water extracts.

The choice of the extraction solvent influences the resulting profile of isolated bioactive compounds present in the extract due to differences in solvent polarity^56–58^. Previously, water and ethanol plant extracts were shown to contain different total phenolic and flavonoid content, which paralleled differences in their antioxidant activities^56,59^. As mentioned earlier, there was a significant disparity between the metabolomic profiles of lyophilized water and aq. EtOH extracts (Fig. 2a). The distribution of metabolites that most contributed to this variability are presented in a PCA loadings scatter plot (Fig. 2b). The different isolated metabolites in each extract have therefore distinct chemical properties and behave differently when exposed to high temperatures. Accordingly, the lower sensitivity to post-extraction drying temperature observed with aq. EtOH extracts suggests that the isolated metabolites using this solvent are more thermally stable. This can also explain the increase in the detected features at 60 °C compared to 40 °C in oven-dried water extract suggesting that the metabolites present in this extract are more prone to undergo structural changes.

### Phytochemical compounds abundance and chemical transformations

Differences in chemical profiles of *A. macrostachyum* water and aq. EtOH extracts after lyophilization and oven-drying were further analyzed by hierarchical clustering analysis (HCA) and Partial Least Square Discriminant Analysis-Variable Importance in projection (PLSDA-VIP) score ranking of the top 50 features most responsible for the observed differences between the treatments. Visualizing the data using heat maps clearly demonstrates variations in the global features and identified molecules between water and aq. EtOH extracts (Fig. 2c,d). A full list of the global features (knowns and unknowns) and their corresponding relative abundance values are presented in (Supplementary Excel sheet). A total of 619 known metabolites were identified in *A. macrostachyum* extracts. Table 1 shows the top 20 most abundant and differentially present identified metabolites in *A. macrostachyum* extracts. Metabolites identified in this table exhibit a variable importance in projection (VIP) score ≥ 1 in their contribution to the variability between lyophilization and oven-drying. Interestingly, the metabolites that participated most in discriminating between samples were between 10 and 20-fold more abundant in the lyophilized extracts compared to the oven dried extracts, suggesting that oven-drying is altering the stability of these molecules. This could be due to the presence of volatile molecules, such as citrate and basellasaponin C, that might have evaporated in the oven. Prolonged exposure to moderate temperatures has been shown to cause thermal degradation of plant metabolites leading to reduced antioxidant activity and phenolic content^60,61^.

**Table 1:**
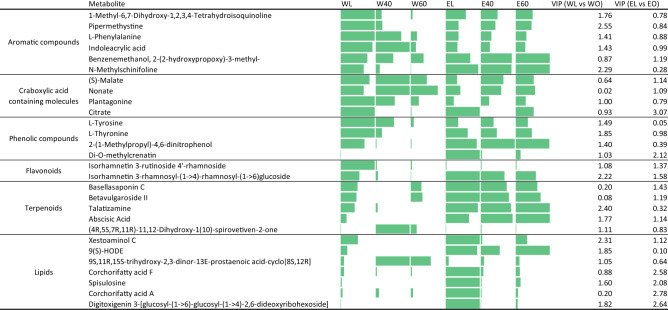
Top 20 most annotated metabolites extracted from *A.macrostachyum* under different conditions and their corresponding abundance in each sample

All the presented metabolites showed VIP score ≥ 1 in the contribution to the variability between lyophilized and oven-dried samples. WL: lyophilized water extract, WO: oven-dried water extract, W40: oven-dried water extract at 40 °C, W60: oven-dried water extract at 60 °C, EL: lyophilized 50% EtOH extract, EO: oven-dried 50% EtOH extract, E40: oven-dried 50% EtOH extract at 40 °C, E60: oven-dried 50% EtOH extract at 60 °C.

The effect of the solvent used in determining the most abundant molecules was also highlighted in Table 1. A larger number of metabolites were found to be enriched in relative abundance in aq. EtOH extracts compared to water extracts. These metabolites mainly included molecules classified as lipids. Oven-drying aq. EtOH extracts resulted in a significant decrease in the abundance of these molecules (VIP scores ≥ 1), except for 9(S)-HODE, a linoleic acid derivative. Lipids are easily oxidized by reactive oxygen species generated under heat, resulting in the production of low molecular volatile aldehydes, alcohols, and hydrocarbons^62^. The effect of heating on the extracted lipids from *A. macrostachyum* was further supported by the increase in abundance of 9S,11R,15S-trihydroxy-2,3-dinor-13E-prostaenoic acid-cyclo [8S,12R] in oven-dried water extracts. This molecule is an eicosanoid that is generated by the oxidation of arachidonic acid or other polyunsaturated fatty acids via non-enzymatic free radical mechanisms^63^.

In addition to lipids, terpenoids were also found to be more abundant in aq. EtOH extracts compared to water extracts. Terpenes exhibited higher thermal stability during oven-drying in aq. EtOH extracts (talatizamine and abscisic acid in Table 1). Those molecules belonged to the diterpenoid and sesquiterpenoid subclasses, which are rich in oxygen functional groups exposing them more to hydrolysis at high temperatures, possibly explaining the loss in their abundance in oven-dried water extracts. An exception to this was the terpene glycoside (4R,5S,7R,11R)-11,12-dihydroxy-1(10)-spirovetiven-2-one), which remained highly abundant in the oven-dried water extract (40 °C).

Phenolic compounds were enriched in relative abundance in lyophilized water and aq. EtOH extracts. Oven-drying had no effect on the abundance of these molecules in aq. EtOH extracts except in the case of the phenyl glycoside Di-O-methylcrenatin, which was significantly less abundant in oven-dried conditions (Table 1). Similarly, the flavonoid glycoside Isorhamnetin 3-rhamnosyl-(1->4)-rhamnosyl-(1->6) glucoside showed decreased abundance in oven dried samples. Previous investigation on the effect of heat treatment on glycosylated phenolics and flavonoids has shown that high temperatures result in the decomposition of these molecules^64^. Lyophilized *A. macrostachyum* water extract was highly rich in aromatic compounds including isoquinoline, alkaloid, aromatic amino acids, indole and hydroquinone which became depleted as a result of oven drying at 60 °C. In contrast, oven-drying at 40 °C and 60 °C had no effect on the abundance of the aromatic molecules in in aq. EtOH extracts.

Amino acids were particularly abundant in lyophilized *A. macrostachyum* water extract, and oven-drying resulted in a decrease in the abundance of those molecules, especially at 60 °C. Heating can cause Maillard reaction between amino acids and reducing sugars producing a complex array of reactive compounds explaining the chemical transformation of these molecules at high temperature^62,63^. Dicarboxylic, tricarboxylic, and pyridine carboxylic acids were also abundant in lyophilized water extract and showed variable abundance in oven-dried extracts. Furthermore, oven drying of water extracts resulted in a general decrease in flavonoids, flavonoid glycosides, phenols, alkaloids, benzoic acid derivatives and phenylpropanoids in comparison to the lyophilized sample. In addition to heat, the chemical transformation of metabolites can be promoted by humidity and oxygen. Open-air-drying can result in moisture uptake which directly affects the velocity of the degradation reactions of plant metabolites^65^. Open-air-drying also leads to the formation of free radical oxygen species that can interact with electron donors such as phenolic compounds leading to their oxidative degradation^66^.

**Figure 3:**
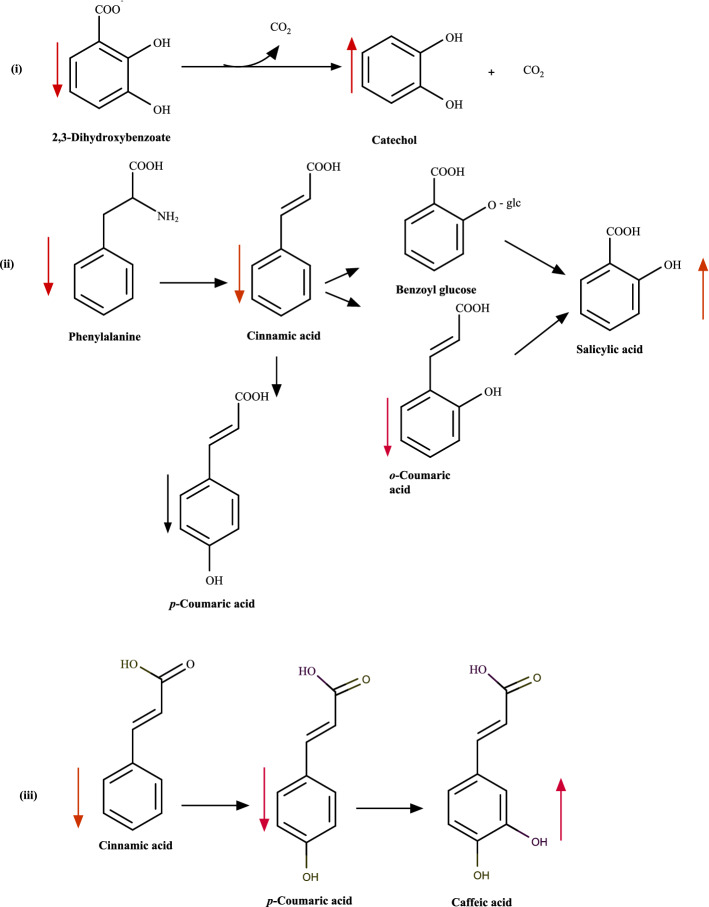
Possible chemical transformations that might have occurred to some of the metabolites of *A. macrostachyum* water and 50% EtOH extracts during oven-drying at 40 °C. Chemical structures were drawn using Marvin 20.21.0 (https://www.chemaxon.com).

Interestingly, these compounds appear to be more resistant to oven drying in aq. EtOH extracts. The evaporation rate of water in water–ethanol mixture is higher than that of pure water, which could explain the stability of metabolites in the aq. EtOH extract^67^. Possible chemical reactions/transformations of some of the *A. macrostachyum* water and aq. EtOH metabolites that are affected by oven drying are illustrated in Fig. 3. Catechol for example was highly abundant in the oven-dried water extract (40 °C) and showed a VIP score > 0.5 in differentiating between the tested conditions. Catechol is formed by the hydroxylation of salicylic acid followed by the decarboxylation of 2,3-dihydroxybenzoate^68^. The precursor molecules involved in this reaction also showed high VIP sores (≥ 0.5) in differentiating between the tested conditions which suggest that these reactions are enhanced during oven-drying at 40 °C. Caffeate which can be generated by the hydroxylation^69^ of p-coumaric acid, was also relatively more abundant in the oven-dried water extract (40 °C) (Supplementary S1). The analysis of our data (Table 1 and Supplementary S1) showed that oven drying at 40 ºC led to ≈ 10- to 20-fold increase (p ≤ 0.001) in the relative abundance of certain metabolites in *A. macrostachyum* water and aq. EtOH extracts in comparison to the lyophilized and 60 °C oven dried extracts. Collectively, the data suggests that more chemical reactions are taking place when extracts are oven-dried at 40 °C compared to 60 °C. Examples of metabolites that were affected by drying at 40 °C are listed in Table 1, and include indoleacrylic acid, (S)-malate, nonate, (4R,5S,7R,11R)-11,12-dihydroxy-1(10)-spirovetiven-2-one and 9S,11R,15S-trihydroxy-2,3-dinor-13E-prostaenoic acid-cyclo[8S,12R] in water extract, and benzenemethanol, 2-(2-hydroxypropoxy)-3-methyl-, 2-(1-methylpropyl)-4,6-dinitrophenol and abscisic acid in aq. EtOH extract.

Pharmacologically relevant molecules that were preserved in oven-dried EtOH extract include abscisic acid (anti-inflamatory)^70,71^, austroinulin (anti-inflammatory properties)^72^, N-methylschinifoline (radiosensitizing properties)^73^, betavulgaroside II (hypoglycemic activity)^74^, talatizamine (protective cardiovascular effect)^75^, and ipratropium (anticholinergic properties)^76^. Like-wise, pharmacologically relevant molecules that were preserved in oven-dried water extracts included the anti-inflammatory indole indoleacrylic acid^70^, and hericenone D, a phenol that is reported to have inhibitory activity against cellular senescence in human umbilical vascular endothelial cells^77^.

### Quantification of phenolic acids

**Figure 4:**
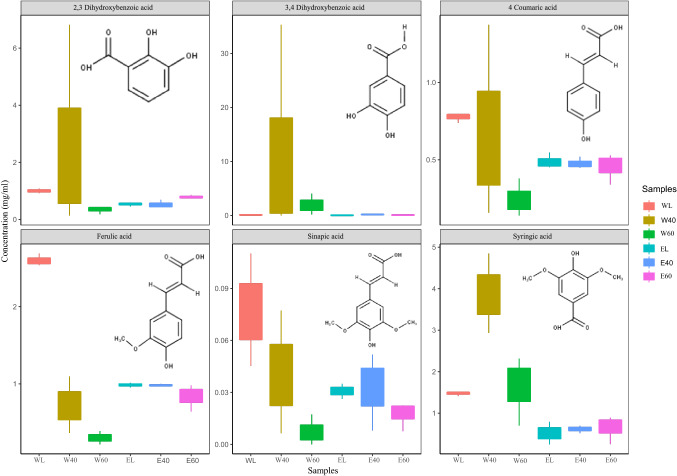
Semi-quantification of phenolic acids in *A. macrostachyum* water and 50% EtOH extracts. Figures displaying semi-quantification were developed using R 3.6.3 (https://www.rstudio.com/products/rpackages/) and ggplot2 package (https://ggplot2.tidyverse.org). Chemical structures depicted were drawn using Marvin 20.21.0 (https://www.chemaxon.com).

To estimate the concentrations of phenolic acids in the *A. macrostachyum* water and aq. EtOH extracts; calibration curves of chemical standards consisting of a mixture of 2,3-Dihydroxybenzoic acid, 3,4-Dihydroxybenzoic acid, syringic acid, 4-coumaric acid, ferulic acid and sinapic acid compared to the abundance in the samples were constructed. The concentrations and structures of the analyzed phenolic acids in *A. macrostachyum* water and aq. EtOH extracts at different drying methods are shown in Fig. 4. Semi-quantification of six phenolic acids was carried for the purpose of assessing the effect of lyophilization and oven-drying at different temperatures on the variability between biological replicates. Semi-quantification of phenolic acid data (Fig. 4) was also compared to the relative abundance of these molecules from the metabolomics data (Supplementary S1) to confirm the validity of using relative abundance values as a tool to assess variability between treatments.

The concentrations of hydroxybenzoic acids across all drying methods ranged between 0.06 ± 0.003 and 12.05 ± 5.1 µg/ml (Fig. 4). The strongest variation was observed for 3,4-Dihydroxybenzoic acid (0.06 µg/ml in the aq. EtOH lyophilized extracts and 12.05 µg/ml in the 40 °C oven-dried water extracts). The concentrations of the other two hydroxybenzoic acids; 2,3-Dihydroxybenzoic acid and syringic acid was also higher in 40 °C oven-dried water extracts and correlated with the corresponding with relative abundance values.

The concentrations of the hydroxycinnamic acids; 4-coumaric acid, ferulic acid and sinapic acid varied between 0.025 ± 0.008 and 0.77 ± 0.03 µg/ml. (Fig. 4) compared to its relative abundance (17,886 – 56,506; Supplementary S1). The highest concentration and relative abundance for the three compounds was observed in the lyophilized water extract while the lowest concentration was detected in the oven-dried 60 °C for both water and aq. EtOH extracts (Supplementary S1).

In general, the average abundance of the tested phenolic acids was higher in lyophilized extract when water is used as a solvent in comparison to aq. EtOH. Oven-drying remarkably increased the variability within a treatment group. This effect was more pronounced in water extracts, especially after drying at 40 °C. This finding could be due to metabolites in the extracts remaining in contact with water for a longer time at 40 °C (72 h) compared to extracts dried at 60 °C (60 h) and the lyophilized extracts (36 h). Indeed, previous reports demonstrated that higher heating temperature and time caused a decline in phenolic acids in citrus peel extract (*Citrus paradisi* Changshanhuyou)^64^. Temperature has a great effect on the solvent–solute interaction, which in turn determines the reactivity and stability of the phytochemicals. For instance, water evaporation at 40 °C is slow, which could promote hydrolysis, oxidation–reduction, hydroxylation, condensations, or decarboxylation reactions^78^. This in turn could lead to increased variations in the chemical composition of the biological replicates.

As observed and discussed above, the abundance of the tested phenolic acids was less prone to thermal-dependent variations in the aq. EtOH extracts. In addition, the biological replicates of oven-dried aq. EtOH extracts displayed smaller variability when compared to water extracts. Overall, our analysis showed that when aq. EtOH is used as solvent, metabolites are not only preserved using post-extraction oven-drying, but that also that oven drying produces extracts that are as reproducible as extracts produced via lyophilization.

## Conclusion

Drying is a crucial step in the preparation of plant extracts to preserve bioactive compounds present in the plant material. In this study we compared lyophilization to oven-drying at two temperatures (40 °C and 60 °C) in two extraction solvents (water and aq. EtOH) by evaluating the metabolomics profile of the resulting dried-extracts. Data gathered in this study suggest that oven-drying could be as effective as lyophilization in isolating pharmacologically relevant metabolites from *A. macrostachyum* when aq. EtOH is used as solvent compared to water. Furthermore, oven-drying of aq. EtOH extracts showed high reproducibility between biological replicates. In contrast, oven-drying significantly altered the composition of metabolites in *A. macrostachyum* extracts when water is used as solvent, possibly due to hydroxylation and oxidation reactions, indicating that lyophilization is better suited for water extracts.

## Materials and methods

### Plant collection

**Figure 5:**
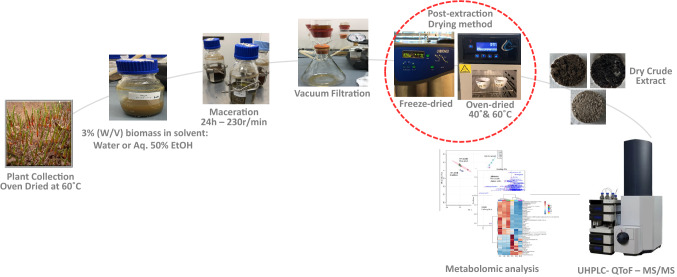
Schematic flowchart of the methodology.

Fresh shoots of *A. macrostachyum* were collected from Al-Maqtaa area, Abu Dhabi, UAE during summer (August). Fresh biomass was oven dried at 60 °C for 24 h. A coffee grinder (Moulinex AR110O27) was used to grind the dry biomass into fine powder. Dry plant material was then stored at room temperature in airtight plastic bags for further analysis. A methodology flowchart is shown in (Fig. 5).

### Plant extraction, and post-extraction drying method

Maceration was used to extract active components from dried and ground *Arthrocnemum macrostachyum* samples. Three grams of biomass were added to 100 ml solvent. Two solvents were used for extraction: water and aqueous 50% ethanol (v/v). The biomass and solvent were macerated for 24 h with a speed of 230 rpm using STIK rotary shaker at room temperature. After extraction, the solvent was separated from the solid biomass via vacuum filtration using Supertek 47 mm filtering glass apparatus, Grade 4. Three batches of filtrates were prepared from each crude extract. The filtrates were either dried in the oven at 40 °C (72 h for water – 60 h for aq. EtOH) or 60 °C (48 h for water – 40 h for aq. EtOH) or lyophilized using LABCONCO FreeZone 4.5 Liter (− 100 °C) Benchtop Freeze Dryer for (36 h for water and aq. EtOH). All extraction and drying steps were performed in triplicate.

### UHPLC-QToF-MS metabolite profiling

Metabolites present in the extracts were analyzed using an Agilent 1290 HPLC system (Agilent, US) coupled to a Bruker Impact II HD Q-ToF-LC/MS (Bruker Daltonics GmbH, Germany). Metabolites were separated using a reversed-phase (RP) separation method. In RP mode, medium-polarity and non-polar metabolites were separated using an Eclipse Plus C18 column (50 mm × 2.1 mm ID) (Agilent, US). Chromatographic mobile phases consisted of MilliQ-H_2_O + 0.2% formic acid (buffer A), Acetonitrile + 0.2% formic acid (Buffer C). The gradient started with 95% A and 5% C, with an initial gradient of 18 min to 100% C and a holding time of 2 min. Every run was followed by a 5 min washing step cycling from buffer C to buffer A to Isopropanol (buffer D) and back to the starting condition, where the column was equilibrated for another 2 min. Detection was carried out in positive and negative ionization modes with the following parameters: ESI settings: dry gas temperature = 220 °C, dry gas flow = 8.0 l/min, Nebulizer pressure = 2.2 bar, Capillary = 4500 V, end plate Offset = (-)500 V; MS-ToF setting: Funnel1 RF = 150 Vpp, Funnel 2 RF = 200 Vpp, hexapole RF = 50, Quadrupole = 1 eV, (Collision Energy for full scan MS = 7 eV, untargeted MSMS = stepping 30–50 eV); Acquisition Setting: mass range = 50–1300 m/z, Spectra rate = 6.0 Hz spectra/s, 1000 ms/spectrum.

### Quantification of phenolic acids

For semi-quantification (quantification without spike of isotope labeled standard in samples to indicate matrix effects) a dilution series for the phenolic acids 2,3-Dihyrdoxybenzoic acid, 3,4-Dihydroxybenzoic acid, 4-Coumaric acid, Ferulic acid, Sinapic acid and Syringic acid (Sigma, GER) was prepared in MilliQ-H_2_O at 0.001, 0.01, 0.1, 1, 2, 4 µg/mL. The mixture of standard dilutions was measured on the above mentioned UHPLC-MS system and data was fitted to a linear calibration curve. Figures displaying semi-quantification were developed using R 3.6.3 (https://www.rstudio.com/products/rpackages/) and ggplot2 package (https://ggplot2.tidyverse.org/). Chemical structures depicted were drawn using Marvin 20.21.0 (https://www.chemaxon.com).

### Data processing

Every spectrum was individually calibrated, aligned with an internal lock-mass and peak picking was performed using the T-Rex 3D algorithm of Metaboscape 4.0 (Bruker Daktronics GmbH, Germany). Background noise was removed by applying an intensity threshold of 2000. Peak-picking and integration was accompanied with ^13^C cluster detection for molecular feature verification.

Annotations were generated by running the molecular features against mass list containing based on metabolites found in KEGG pathways within a mass difference of 5 ppm. These annotations were verified by their isotopic pattern (sigma factor below 3). Finally, the putative annotations were compared with a fragmentation library consisting of the IROA metabolite library (IROA Technologies, USA) and various standards (~ 800 molecules) measured in house and the Bruker Personal Library (15,000 Molecules, 65,000 MSMS Spectra). Molecules with a MSMS match factor of > 800 (max. 1000) were deemed verified metabolites.

### Multivariate data analysis

Total area normalization was performed on the filtered data to reduce the systematic biases within the samples. All variables were log transformed and Pareto scaled for multivariate statistical analysis to remove the offsets and adjust the importance of high and low abundance metabolites to an equal level (principal components analysis (PCA), partial least squares discriminant analysis (PLS-DA), and hierarchical cluster analysis (HCA). Multivariate statistical analysis was performed using MetaboAnalyst 4.0. PCA plots were used to illustrate the distribution of the original data. Heat maps were constructed by applying Euclidean distance measurements and ward clustering algorithm to obtain a PLSDA-VIP scores for the top 50 features responsible for difference between samples using MetaboAnalyst 4.0. based on PLSDA-VIP fitted model. The PLS-DA was validated by the sevenfold cross validation and permutation test (200 permutations). The PLS-DA model was used with the first principal component of VIP (Variable Importance in Projection) values combined with Student’s t-test to determine significantly differentially abundant metabolites in water and 50% EtOH *A. macrostachyum* extracts. Multi-criteria assessment (MCA), including the variable importance in projection (VIP) values and p values, were used to screen and select the potential metabolites. The MCA was performed using the following statistical criteria: VIP > 1, and 3. *p* value < 0.05. All the results are presented as means ± SE.

## Supplementary Information

Supplementary Information 1.

Supplementary Information 2.
